# Supplementary material for: Effect of the Composition of Copolymers Based on Glycidyl Methacrylate and Fluoroalkyl Methacrylates on the Free Energy and Lyophilic Properties of the Modified Surface
Source: Polymers (Basel). 2022 May 11;14(10):1960. doi: 10.3390/polym14101960 (PMC9145383; doi:10.3390/polym14101960)
Supplement: Supplementary file 1 [file polymers-14-01960-s001.zip › figure S1, table S1, table S2.pdf]

Table S1 Work of adhesion of the test liquids (water, n-decane, diiodomethane) on the surface of glasses modified with copolymers of glycidyl metharylate and fluoroalkyl methacrylates depending on the content of FMA

Modifier	Molar content [FMA], %	Work of adhesion (W_{sl}), mN/m					
		Calculated by the Young-Dupre formula			Calculated in DataPhysics SCA 20 software		
		Water	n-decane	Diiodomethane	Water	n-decane	Diiodomethane
Poly-GMA	0	94,4±1,3	47,0±0,1	93,5±1,0	95,0±1,2	58,3±0,4	83,9±0,7
Poly-(TEMA-co-GMA)	33,3	64,1±1,4	44,8±0,3	75,5±0,3	64,3±1,3	51,9±0,1	71,5±0,4
	50,0	63,5±1,4	44,1±0,1	68,1±0,3	63,5±1,4	48,6±0,1	67,6±0,4
	66,7	59,2±1,5	43,9±0,2	67,8±0,4	59,3±1,5	48,6±0,2	66,8±0,4
Poly-TEMA	100	55,5±1,5	43,2±0,1	67,2±0,4	55,4±0,5	43,1±0,2	64,9±0,4
Poly-(HFMA-co-GMA)	33,3	69,8±0,2	47,0±0,1	78,3±0,8	69,9±1,3	49,1±0,2	74,9±0,5
	50,0	62,1±0,1	44,4±0,1	67,0±0,5	62,1±1,1	44,2±0,2	67,3±0,5
	66,7	57,3±1,0	42,1±0,2	58,0±0,6	57,1±1,0	40,1±0,2	61,2±0,5
Poly-HFMA	100	54,5±0,7	40,9±0,3	55,7±1,1	54,3±1,1	38,8±0,4	59,0±0,6
Poly-(HIMA-co-GMA)	33,3	60,3±1,4	42,3±0,6	60,8±1,7	60,2±1,2	41,0±1,0	62,8±1,5
	50,0	56,1±1,1	41,5±0,3	57,5±1,1	55,9±1,2	39,6±0,5	60,4±0,7
	66,7	47,6±1,1	41,0±0,3	54,8±1,3	47,5±1,0	38,9±0,8	58,1±1,0
Poly-HIMA	100	47,3±2,0	39,0±0,2	53,9±1,2	47,1±1,8	37,5±0,5	56,2±0,9
Poly-(HBMA-co-GMA)	33,3	53,6±0,5	39,5±0,5	54,3±0,8	53,5±0,5	37,6±0,4	57,3±0,7
	50,0	51,0±0,7	39,1±0,4	54,2±0,4	50,9±0,8	37,5±0,3	56,8±0,4
	66,7	47,5±0,9	37,4±0,3	47,5±1,2	47,2±1,0	34,5±0,5	52,3±0,8
Poly-HBMA	100	46,3±0,9	36,8±0,2	49,3±0,3	45,2±1,0	34,8±0,1	52,5±0,3

Table S2 Work of adhesion of the test liquids (water, diiodomethane) on the surface of textured aluminum samples modified with copolymers of glycidyl metharylate and fluoroalkyl methacrylates depending on the FMA content

Modifier	Molar content [FMA], %	Work of adhesion (W_{sl}), mN/m			
		Calculated by the Young-Dupre formula		Calculated in DataPhysics SCA 20 software	
		Water	Diiodomethane	Water	Diiodomethane
Poly-GMA	0	13,66	81,02	93,59	93,47
Poly-(TEMA-co-GMA)	33,3	4,49	4,91	4,94	4,99
	50,0	3,16	4,91	3,00	4,87
	66,7	1,58	2,85	1,73	2,81
Poly-TEMA	100	1,59	2,51	1,50	2,44
Poly-(HFMA-co-GMA)	33,3	2,87	2,85	3,19	3,04
	50,0	2,30	2,60	1,50	2,44
	66,7	2,12	2,09	1,22	1,99
Poly-HFMA	100	1,40	2,07	1,22	1,99
Poly-(HIMA-	33,3	2,06	2,74	1,73	2,81
	50,0	1,85	2,40	1,50	2,44

co-GMA)	66,7	1,33	2,12	1,22	1,99
Poly-HIMA	100	1,28	2,07	1,22	1,99
Poly-(HBMA-co-GMA)	33,3	1,66	2,46	1,50	2,44
	50,0	1,38	2,19	1,22	1,99
	66,7	1,33	2,02	1,22	1,99
Poly-HBMA	100	1,10	2,02	1,22	1,99

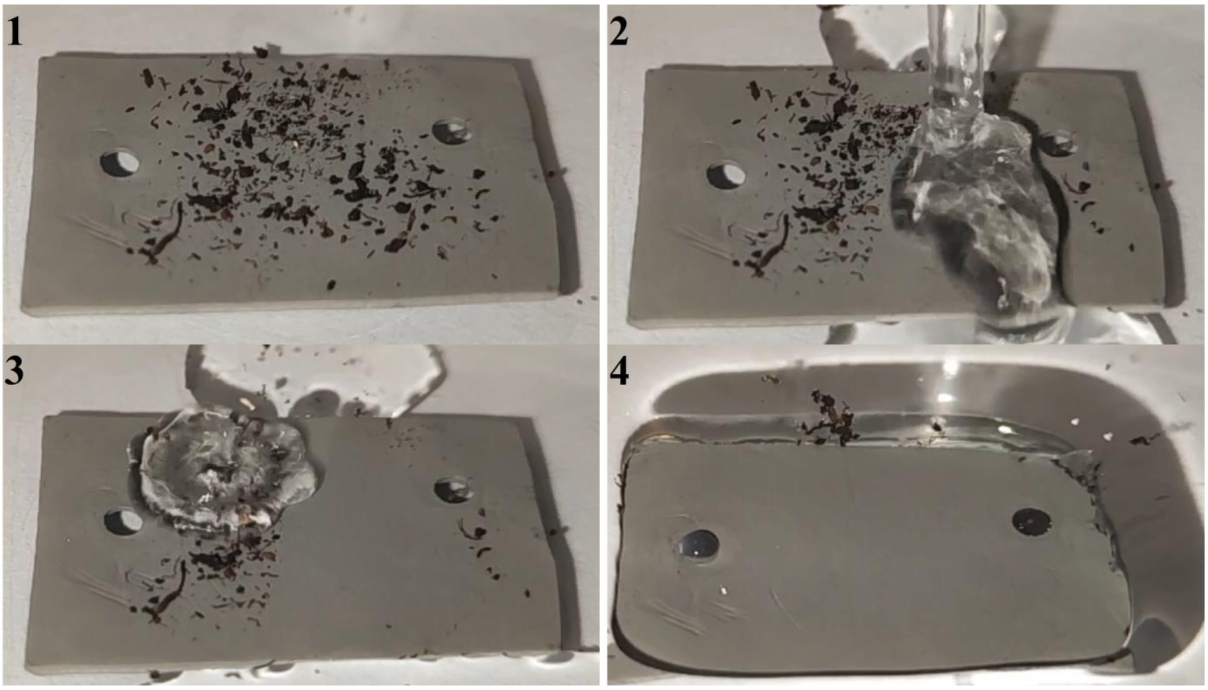

Figure S1 Demonstration of the self-cleaning effect of the surface of superhydrophobic textured aluminum as a result of the action of a jet of water and large droplets
